# Gonadal Sex and Sex-Chromosome Complement Interact to Affect Ethanol Consumption in Adolescent Four Core Genotypes Mice

**DOI:** 10.3390/brainsci16060597

**Published:** 2026-05-30

**Authors:** James D. Jentsch, Shawn M. Aarde, Jared R. Bagley

**Affiliations:** Department of Psychology, Binghamton University, Binghamton, NY 13902, USAjbagley@binghamton.edu (J.R.B.)

**Keywords:** four core genotypes, drinking-in-the-dark, bingeing, adolescent, alcohol, ethanol, sex differences, sex-chromosome complement

## Abstract

**Highlights:**

**What are the main findings?**

- In adolescent four core genotypes mice, gonadal males drink more ethanol than gonadal females, particularly in the presence of an XX karyotype, and XY mice drink more ethanol than XX mice, but only in gonadal females.

**What are the implications of the main findings?**

- The effects of sex-biasing biological factors on the patterns of ethanol consumption by adolescent four core genotypes mice that we observed in our 3-bottle drinking procedure showed face validity with some of the sex/gender differences observed in human adolescents.

**Abstract:**

Background/Objectives: Sex differences in ethanol consumption have been reported in both humans and laboratory rodents, but the independent/dependent contributions of genetic and hormonal sex biasing mechanisms to these phenotypes have not yet been fully explored. Methods: To examine the contributions of sex-chromosome complement (SCC) and gonadal sex (GS) to ethanol consumption, we studied adolescent (28–32 days old) four core genotypes (FCG) mice on a C57BL/6J background, a model which allows for independent assortment of GS and SCC. A modified drinking-in-the-dark (DID) procedure was employed, in which mice were offered concurrent access to 20%, 10% and 0% ethanol (in water) in four daily 2 h sessions. Consumption at the level of individual bouts was recorded. Results: Overall ethanol intake differed substantially by group and was driven almost entirely by differences in consumption of the 20% ethanol solution; all groups preferred the 20% solution over the 10% and 0% solutions, but consumed similar amounts of the 10% and 0% solutions. Intake of the 20% ethanol solution followed the rank order XXM > XYM > XYF > XXF. This pattern reflects an interaction between SCC and GS, such that SCC effects were greatest in gonadal females (XY > XX), whereas GS effects were greatest in XX mice (gonadal males > gonadal females). Moreover, the magnitude of these effects varied both across and within drinking sessions. The behavioral microstructure of ethanol consumption (i.e., parameterization of within-session discriminable drinking bouts) support the validity of our three-bottle modification of the DID procedure as a model of binge-like consumption, because (1) the consumption rate of the 20% ethanol solution was ~80 g EtOH/kg/h within a bout (~12 s/bout, ~three bouts/session), (2) most of this ethanol consumption was completed in a single bout and (3) within-session ethanol consumption was greater earlier than later, indicating “front loading.” Conclusions: These results suggest that the effects of GS on binge-like ethanol consumption are observed in early adolescence and moderated by SCC in adolescent FCG mice, with GS effects most pronounced in XX mice and SCC effects evident mainly in gonadal females.

## 1. Introduction

Biological sex has been associated with differences in patterns of adolescent alcohol (ethanol) consumption (e.g., binge frequency, age of first experience and rate of progression, motivations to drink, and reactivity to positive/negative experiences), as well as to alcohol use disorder (AUD) [1,2,3,4,5,6,7,8,9,10,11]. These observations implicate effects of, and interactions between, gonadal hormones [12,13,14,15,16,17,18], gene–sex interactions [19,20,21,22,23,24] and/or gendered socio-cultural factors [6,25,26] on responsivity to ethanol.

Of particular concern is adolescent binge drinking, which is defined as the consumption of enough ethanol within 2 h to achieve blood ethanol concentrations (BECs) ≥ 80 mg/dL [27], as it predicts future alcohol dependence and AUD [1,28,29,30,31,32]. It has been estimated that ~77% of the costs associated with alcohol use in the U.S. are attributable to binge drinking [33]. Sex differences in alcohol drinking increase from 8th to 12th grade, by which stage bingeing is substantially greater in males (16%) than females (12%) [5,34]. Although this sex difference continues into adulthood, its magnitude has been decreasing over time, largely due to increasing alcohol use by females [35,36,37].

Sex differences in phenotypes such as binge drinking may arise from three major sex-biasing factors: (1) long-lasting organizational effects of gonadal hormones that are established during critical developmental periods, (2) activational effects that depend on circulating gonadal hormones in adulthood and diminish upon insufficient hormone levels, and (3) SCC mechanisms, which reflect differences in X- and Y-linked genes and their dosage [38,39]. People with an XY karyotype typically have a functional *SRY* (sex determining region of the Y chromosome) gene that guides gonad differentiation toward the development of testes, or sans *SRY*, toward the development of ovaries, which subsequently determines the type of gonadal hormones secreted [40,41]. These coupled mechanisms make it difficult to fully dissect the causal effects of, and interactions between, these three sex-biasing factors.

However, animal models are available that enable decoupling the *Sry* gene from the Y-chromosome. For example, in the FCG mouse, the *Sry* gene has been deleted from the Y chromosome and is instead expressed from an autosomal transgene [42,43,44,45]), thereby making GS independent from SCC and resulting in four types of individuals: mice with an XX karyotype that have ovaries or testes (termed XXF or XXM, respectively) or with an XY karyotype that have ovaries of testes (termed XYF or XYM, respectively). Research using this model has shown direct genetic effects of SCC on adult ethanol consumption [19] and on the formation of habitual operant responding for ethanol [20]. Moreover, the importance of both the direct effects of SCC and its interactions with GS on basic reward/reinforcement processes, as well as cognitive functions that are centrally implicated in AUD and other addictions, has been underscored by both human studies on sex-chromosome aneuploidies [46,47] and in animal models like the FCG mouse [48,49,50,51,52,53,54] and those that model sex-chromosome aneuploidies [55,56,57,58,59,60,61,62].

Thus, discovering and validating useful models of sex-chromosome effects on alcohol consumption and AUD would greatly benefit from testing a preclinical animal model like the FCG mouse using a procedural model of ethanol bingeing behavior. One such model that has demonstrated construct and predictive validity in the FCG mouse background strain C57BL/6J is the drinking-in-the-dark (DID) model established by Rhodes et al. [63,64,65,66,67]. The key feature of this procedure is the circadian placement and duration of ethanol access: mice are provided with limited (2–4 h) access to an ethanol solution (typically 20%) in their home cage, starting 3 h after the onset of the animals’ dark phase. Binge-like ethanol intake was confirmed by BECs that regularly exceeded 100 mg/dL. Moreover, studies of DID that include behavioral microstructural intake data (i.e., parameterized high temporal resolution behavioral recordings) have similarly shown that mice concentrate ethanol consumption into short discriminable binge-like bouts [67,68,69] and that sex differences can be detected in both these bout-level measures [67,68] and within-session changes in intake (e.g., typically greater “front loading” in females) [70,71].

Importantly, although sex differences observed in DID-based procedures typically show greater ethanol consumption in female mice than male mice [72,73,74,75,76,77,78], these sex differences are sometimes not detected [79,80,81,82,83], vary from one experiment to the next within a report [64,67,70,84] and/or show greater intake in males than females [69]. This suggests that some procedural factors that vary across research studies moderate sex effects on ethanol drinking behaviors.

Additionally, using a non-DID drinking procedure, Sneddon et al. [20] studied adult FCG mice that were provided access to increasing concentrations of ethanol (5–>10–>15–>20%; clusters of 5 consecutive sessions/concentration at 24 h/session) and water. Group differences in ethanol consumption were concentration dependent, as XX mice consumed more ethanol than XY mice for only the 15% solution (similarly for preference; XX > XY only at 15% ethanol), and gonadal females consumed more ethanol than gonadal males for only the 20% solution (no GS differences in preference were detected). Thus, one way to maximize the likelihood of detecting the effects of SCC, GS and their interaction in the DID procedure may also be to include access to a range of concentrations.

Because there is no research addressing the role of biological sex-biasing factors in adolescent ethanol consumption, we sought to examine voluntary drinking in adolescent, gonad-intact FCG mice with a DID procedure that provided mice with access to water and two ethanol concentrations (10 and 20%) and incorporated near-real-time measures of drinking behavior that allowed for the measurement of individual profiles of drinking, including front-loading behavior. We hypothesized that gonadal females would drink more ethanol than gonadal males and would do so by engaging in smaller but more frequent consumption bouts [68,69]. Additionally, we predicted greater ethanol consumption by XX mice than XY mice [20]. Lastly, we predicted an SCC*GS interaction such that the SCC effect would be greater in gonadal males than gonadal females [20], and the GS effect would be greater in XX mice than XY mice [19].

## 2. Materials and Methods

### 2.1. Subjects

Subjects were early adolescent mice (28–29 days old at DID start) from the FCG model [85]; FCG mice were originally maintained on an MF1 background before full backcrossing to the C57BL/6J genetic background. In the FCG model, the Y chromosome is deleted for *Sry* (called the Y^−^ chromosome), and *Sry* is introduced onto chromosome 3 as a transgene (Sry+) [45]. XY^−^(Sry+) sires mated with XX(Sry−) dams produced progeny of four genotypes: XY^−^(Sry+) with testes, XX(Sry+) with testes, XY^−^(Sry−) with ovaries and XX(Sry−) with ovaries. While recognizing that biological sex cannot be reduced simply to gonadal type, we describe Sry− mice (i.e., with ovaries) as gonadal females (F) and Sry+ mice (i.e., with testes) as gonadal males (M), for simplicity and consistency with prior literature. Thus, we refer to the four genotypes as XXF (XX + ovaries; *n* = 39), XXM (XX + testes; *n* = 39), XYF (XY + ovaries; *n* = 38) and XYM (XY + testes; *n* = 35). Genotypes were determined via standardized probes for both Y^−^ and *Sry* via genotyping services provided by Transnetyx (Transnetyx, Inc., Cordova, TN, USA) and confirmed by gonadal phenotype [44]. Procedures and protocols were approved by the Institutional Animal Care and Use Committee (IACUC) at Binghamton University and were carried out in a manner consistent with the “Guide for the Care and Use of Laboratory Animals, Eighth Edition” [86].

The mice studied here were drawn from 29 litters each born to a unique dam; the average litter size was 5.2 (range 1–10).

Mice were group housed (2–4 mice/cage of the same GS) in standard open-top home cages with woodchip bedding (to minimized phytoestrogens/pseudoestrogens found in corncob bedding [87]), ad libitum chow (PicoLab^®^ Laboratory Rodent Diet 5LOD; LabDiet, Inc., St Louis, MO, USA; note that as this formulation includes soybeans, it may contain the phytoestrogen genistein) [88], and environmental enrichment that included a nesting pack (~2” × 2” paper pillow stuffed with strips of paper), a small wood block and a red plastic tube. Subjects were kept under a 12:12 light/dark cycle (lights off at 10 AM) in a temperature-controlled (~72 °F) and humidity-controlled (~46%) vivarium.

### 2.2. Drinking-in-the-Dark Procedure

#### 2.2.1. Equipment

Liquid (i.e., ethanol solution) consumption was monitored using a 3-bottle apparatus attached to a modified standard home cage, as described previously [89]. Bottles sit on a load cell that measures small changes in bottle weight and contact by the animal, at a sampling rate of ~2.2 samples/second. Thus, load cells serve as contact sensors and mass scales that permit high-resolution discrimination of both bout frequency/duration and amount consumed per bout. Bottles were constructed from modified 50 mL conical tubes fitted with a silicone stopper and a curved sipper tube, within which two steel ball bearings were placed to minimize non-contact liquid loss. As the temporally discrete recording of consumption includes a taring and re-taring of bottle weight, the contamination of non-contact liquid loss (e.g., “leak”) is minimized. Test cages were placed within sound- and light-attenuating cubicles equipped with a fan for ventilation and white noise. Each cage contained a fresh layer of woodchip bedding and was topped with a standard wire cage lid.

During each daily session, one monitor was equipped with solution-filled bottles but had no animal placed within it; this unit was used to quantify weight changes in the bottles attributable to leakage, and values from the chambers containing animals were adjusted based on these measurements. Additionally, bottles were weighed individually on a calibrated balance before and after each session, as a comparison to the computer-calculated weights described above. As reported earlier [89], these values precisely tracked one another, showing that the automatic measurements in our devices reliably correspond to actual consumption-related bottle weight changes.

#### 2.2.2. Habituation Phase

On the day prior to the first DID session, mice were habituated to handling (weighing and tail marking), transport to the testing room, the testing room environment (all electronic equipment on), and ethanol odor/taste by dropping ~1 mL of 20% ethanol on their home cage nesting pack.

#### 2.2.3. DID Testing Phase

The day after habituation, mice were tested for liquid consumption over four daily 2 h DID sessions that began 2 h after the start of the dark phase of their light cycle on each of 4 consecutive days. Three bottles of different ethanol concentrations (20%, 10%, and 0%; in tap water) were provided, as was a ~4 g food pellet; the position of the three solutions relative to one another was systematically varied across days so that position was never consistently associated with a particular concentration between mice or across days. Two to three h before each session, mice were weighed, tail-marked and transported to the testing room. Mice were weighed again immediately after each session and returned to the vivarium within 30 min. To minimize isolation stress, mice were only singly housed during DID sessions.

#### 2.2.4. Blood Ethanol Concentrations (BECs)

To estimate the approximate BECs of mice immediately after DID sessions, serum from trunk blood was collected from a subset of mice within 5 min of the end of the last DID session and stored at −80 °C until being analyzed with an Analox system (Analox Instruments Ltd., Stourbridge, UK; AM1 Alcohol Analyzer using a 100 mg/dL calibration standard and available quality control serum [GMRD-110D4]). This group included mice from all 4 genotypes (XXF, *n* = 11; XYF, *n* = 6; XXM, *n* = 7; XYM *n* = 9).

#### 2.2.5. Measures, Design and Analyses

Behavior was recorded as a sequence of discriminable bouts of liquid consumption, as described previously [89]. To filter out spout contacts that were not associated with drinking, bouts were delimited by pauses of ≥5 s (limit of temporal resolution) and those of amounts smaller than 5 mg were excluded, as such values approximate a limit of detection (non-consummatory contacts are distributed around zero within a ±5 mg range). Imposing this 5 mg threshold resulted in a minimal reduction of total weight consumed per bottle (1.6 mg on average) over the 2 h session and reduced contact bouts/time to a greater degree (a decrease of 22 bouts and 159 s), indicating that the threshold largely eliminates non-consumption contacts. Three per-bout measures were recorded—amount, duration and rate (here, rate is expressed as amount [g/kg] per hour even though individual bouts typically lasted <1 min). Using high-resolution bout data, drinking behavior within each session was parameterized to better estimate within-session behavioral dynamics. These consumption parameters included the following: counts of bouts, sums of bout amounts and durations, maximum bout amount and duration, mean and maximum within-bout rate, and latency to 1st bout. All parameters were calculated as per-session measures. Bout counts, sums of bout amounts and durations, and mean within-bout rates, were additionally parsed into 40 min time bins, which were collapsed across sessions to examine within-session changes in consumption.

Measures of liquid consumption amount and rate were normalized to body weight (BW), and ethanol consumption amount and rate were estimated from the combined consumption of 20% and 10% ethanol solutions, adjusted for the density of the solutions (20%EtOH × 0.166 + 10%EtOH × 0.166/2). Mean unit imputation was used to replace missing values due to a software error that caused the loss of data from one session of 3 mice.

### 2.3. Statistical Analyses

Behavioral measures were analyzed using generalized linear mixed models (GLMMs) implemented in SPSS (IBM, Armonk, NY, USA, version 28). GLMMs were selected because they readily accommodate hierarchical data structures, including the inclusion of litter as a random effect to account for within-litter (intra-cluster) correlation [90], and provide greater power than traditional ANOVA-based approaches for repeated-measures designs [91,92,93].

In addition, GLMMs in SPSS allow flexible specification of outcome distributions (normal, gamma, or inverse Gaussian) and link functions, which define the relationship between the linear predictor and the outcome variable and obviate the need for non-linear transformations of the raw data [94]. Degrees of freedom were estimated using the Satterthwaite approximation, which is appropriate for unbalanced designs with complex covariance structures. Diagonal covariance structures were used for repeated measures, variance-components covariance structures were used for random effects, and robust estimation of fixed effects and coefficients was applied to mitigate potential violations of model assumptions.

Preliminary analyses using GLMMs were conducted to determine the best-fit distribution and link function by comparing corrected Akaike Information Criterion (AICc) values of models that included only the intercept and a random litter effect (subject block and intercept). A large proportion of data values were zeros, and thus a constant was initially added to the raw data to test gamma and inverse Gaussian distributions. However, as the magnitude of this constant was associated with large variations in AICc [90], all final results used only the raw data with normal distribution and a link function that produced the smallest AICc (the link functions compared were limited to a Tukey’s ladder of powers: values of 2, 1.5, 1 [identity], 0.5, 0.25, “0” [log10], −0.25, −0.5, −1, −1.5, and −2) excluding those link functions that produced errors. Graphs depict the raw data.

As the detection of interactions was the focus of this study, and as the power to detect interactions is reduced relative to main effects in a full-factorial model [91,92,93], we also report results from simplified models (e.g., interaction term only) where they were informative (e.g., effect-size estimates). Importantly, interaction-only models are simply reparameterizations of their respective full-factorial model (same overall model degrees of freedom, AICc and post hoc values for sequential Sidak corrected simple effects; lower-level parameters are moved to higher-level interaction terms; thus, there are higher degrees of freedom and increased power for those higher-level terms).

Significant omnibus effects and planned comparisons were delineated using post hoc paired-means comparisons in SPSS, with the estimated means function of the GLMM and a sequential Sidak correction for multiple comparisons (post hoc *p*-values represent Sidak-corrected values). Cohen’s f2 was used as a measure of effect size for omnibus fixed effects [94], and Cohen’s d was used as a measure of effect size in paired-means comparisons; calculated respectively by the F_to_f2 and t_to_d functions of the effect size package R (The R Foundation for Statistical Computing, version 4.1.3). Conventions for effect size magnitudes of “Large”, “Medium” and “Small” are ≥0.35, ≥0.15, and ≥0.02 for Cohen’s f2 and ≥0.8, ≥0.5, and ≥0.2 for Cohen’s d [16]. Ancillary measures (pre-session body weight, % post-session body weight loss, food consumed, solution preferences, and blood ethanol concentrations; collapsed across session and time bin) were analyzed with bootstrapped ANCOVA with Litter as a random factor due to failures of matrix convergence for Litter with the GLMM. Correlation tests were 2-tailed non-parametric Spearman’s rho with a false discovery rate correction [95].

## 3. Results

### 3.1. Liquid Consumption Parameters: SCC and Gonadal Effects as a Function of Ethanol Concentration

**Figure 1:**
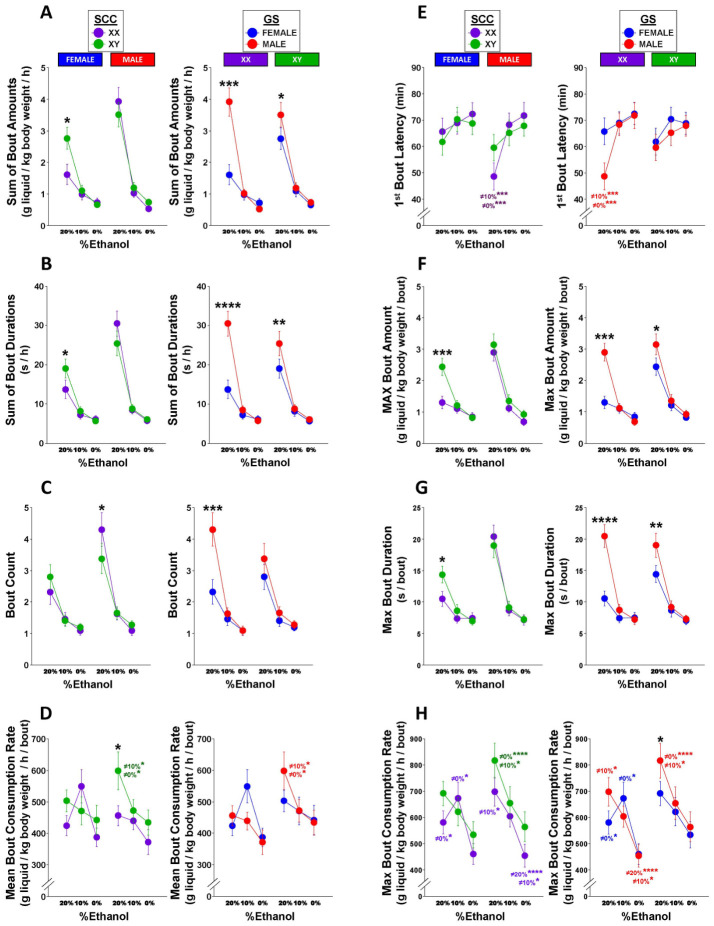
Mean per-session bout Parameters as a function of ethanol concentration. Figures plot mean values (error bars represent ±SEM) of per-session bout parameters for each ethanol concentration (20%, 10% or 0%) that are grouped by GS (FEMALE [i.e., with ovaries] or MALE [i.e., with testes]) and split SCC: XX or XY) (left side of each letter) or vice versa (right side of each letter) so as to better visualize simple effects of GS and SCC within levels of the 2nd genotype factor. These parameters include the Sum of Bout Amounts ((**A**), g/kg/h), Sum of Bout Durations ((**B**), s/h), Bout Count (**C**), Mean Bout Consumption Rate ((**D**), g/kg/h/bout; within-bout rates), 1st Bout Latency ((**E**), minutes from session start), Maximum Bout Amount ((**F**), g/kg/h/bout), Maximum Bout Duration ((**G**), s/h/bout), and Maximum Bout Consumption Rate ((**H**), g/kg/h/bout; within-bout rate). Sidak-adjusted significance values for simple effects of SCC and GS: * *p* < 0.05, ** *p* < 0.01, *** *p* < 0.001, **** *p* < 0.0001. Sidak-adjusted significance values for simple effects of ethanol concentration within genotype use the same asterisk coding for *p*-values, but are color coded to indicate genotype group and indicate ethanol concentration value from which that mean differs (e.g., ≠10%* in blue represents a significant difference from the 10% ethanol solution in females). For clarity, the simple effects of ethanol concentration within genotype are not included for bout amounts (sum and max), bout durations (sum), and bout counts, as all *p* < 0.05 except for the following: XXF 10% vs. 0% for sum of bout amounts (*p* = 0.1) and durations (*p* = 0.2), and for all simple effects in maximum bout amount (all *p* > 0.08) and bout duration (all *p* > 0.05); other 3 genotypes 10% vs. 0% for maximum bout duration (all *p* > 0.06). Group n values (O = ovaries, T = testes): XXF = 39, XXM =39, XYF = 38 and XYM = 35.

Liquid consumption (sum of the amounts of all measured bouts; g liquid/kg BW/h) was ~3× greater for 20% ethanol (M = 2.9) than for either 10% (M = 1.1) or 0% (M = 0.7), indicating a strong ethanol preference (*p* < 0.000001; f2 = 1.91) (Figure 1A). An SCC*GS*liquid type interaction was confirmed (*p* = 0.001, f2 = 0.07). For 20% ethanol, the group rankings for ethanol bout amounts were: XXM (M = 3.9) > XYM (M = 3.5) > XYF (M = 2.7) > XXF (M = 1.6) (simple effects: XYF > XXF, XXM > XXF, XYM > XYF; all *p* < 0.05) with no SCC or GS differences in 10% or 0% ethanol consumption (all *p* > 0.10).

Similar patterns were observed in the sum of bout durations (Figure 1B); there was a ~3× greater consumption time for 20% ethanol (M = 22) than for either 10% (M = 8) or 0% (M = 6) (*p* < 0.000001; f2 = 1.39), and group rankings for 20% ethanol consumption duration of XXM (M = 30) > XYM (M = 25) > XYF (M = 19) > XXF (M = 14) (SCC*GS*liquid type: *p*(full) = 0.11, f2(full) = 0.02, *p*(3-way only) < 0.000001, f2(3-way only) = 2.39) (simple effects: XYF > XXF, XXM > XXF, XYM > XYF; all *p* < 0.05).

As expected, bout amount and duration were tightly correlated for 20% ethanol (rs(151) = 0.95, *p* < 0.000001), 10% ethanol (rs(151) = 0.90, *p* < 0.000001) and 0% ethanol (rs(151) = 0.86, *p* < 0.000001). Thus, the additional variance in bout amount introduced by normalizing to BW did not affect the tight correlation between bout amount and bout duration. This indicates that observed group differences in consumption are unlikely to be attributable to differences in BW.

Although the total number of drinking bouts per session (M = 6) was low compared to the maximum possible (720), values were similar to those previously reported using both bottle-weight measures [69] and conventional lickometers [68] (Figure 1C). The patterns of bout counts across ethanol concentrations and between genotypes mirrored those of bout amounts and durations. A greater number of bouts was observed for 20% ethanol (M = 3.2) than for either 10% (M = 1.5) or 0% (M = 1.2) (*p* < 0.000001; f2 = 1.03), and 20% ethanol bout number rankings were XXM (M = 4.3) > XYM (M = 3.4) > XYF (M = 2.8) > XXF (M = 2.3) (SCC*GS*liquid type: *p*(full) = 0.056, f2(full) = 0.02, *p*(3-way only) < 0.000001, f2(3-way only) = 3.02) (simple effects: XXM > XYM and XXM > XXF; both *p* < 0.05).

Mean consumption rate within bout (g liquid/kg BW/h/bout) increased as ethanol concentration increased (20% M = 493, 10% M = 483, 0% M = 409) (*p* = 0.009, f2 = 0.29) (Figure 1D). However, this pattern varied greatly among genotypes (*p*(full) = 0.19, f2(full) = 0.06, *p*(3-way only) < 0.000001, f2(3-way only) = 1.80). Although XYM mice showed a clear dose-response effect (20% > 10%, 20% > 0%; both *p* < 0.05), the other three genotypes did not (all *p* > 0.13). Lastly, the consumption rate of 20% ethanol was significantly greater for XYM (M = 599) than XXM (M = 456) (*p* = 0.02).

Importantly, these seemingly extreme consumption rates (~450 g/kg BW/h/bout) are due to the high resolution of discriminable short-duration consummatory bouts (i.e., “binges”) while excluding the long pauses between each bout that are often included in calculations of consumption rate (e.g., by simply dividing the total amount consumed in a session-by-session duration). With an average bout duration of ~12 s and bout amount of ~1.5 g/kg BW, the consumption rate during a bout is ~80 g ethanol/kg BW/h for the 20% solution. This microstructural analysis indicates that, in the DID procedure, FCG mice voluntarily consume liquids in a “binge-like” manner (high rate/bout, a few short bouts per hour).

The average latency (min) of the first bout was shorter for 20% ethanol (M = 59) than either 10% (M = 68) or 0% (M = 70) (*p* = 0.00002; f2 = 0.10) (Figure 1E). The first bout latency for 20% was shorter for XXM (M = 49) than for the other three genotypes (XYM M = 60, XYF M = 62, XXF M = 66) (XXM < XXF, *p* = 0.07).

For 20% ethanol, the per-session values for the maximum single-bout amount (M = 2.4 g/kg/bout; Figure 1F) and maximum single-bout duration (M = 16 s/bout; Figure 1G) were only slightly smaller than the per-hour sum of bout amounts (M = 2.9 g/kg/h) and sum of bout durations (M = 22 s/h), indicating that mice drink most of their 20% ethanol in a single large bout. Moreover, the maximum within-bout rate (612 g/kg/h/bout; Figure 1H) was considerably larger than the mean within-bout rate (462 g/kg/h/bout). These observations (and the results below) again indicate binge-like ethanol consumption.

The pattern of ethanol concentration effects and genotype differences for the maximum bout amount (Figure 1F) was similar to that of the sum of bout amounts. Larger maximum bout amounts (g/kg/bout) were observed for 20% ethanol (M = 2.4) than either 10% (M = 1.2) or 0% (M = 0.8) (*p* < 0.000001; f2 = 1.15), and the genotype rankings for 20% ethanol were XYM (M = 3.1) > XXM (M = 2.9) > XYF (M = 2.4) > XXF (M = 1.3) (SCC*GS*liquid type: *p* = 0.0008, f2 = 0.07; simple effects: XYF > XXF, XXM > XXF, XYM > XYF; all *p* < 0.05).

Similarly, the pattern of ethanol concentration effects and genotype differences for the maximum bout duration (Figure 1G) was similar to that of the sum of bout durations. Larger maximum bout durations (s/bout) were observed for 20% ethanol (M = 16) than either 10% (M = 8) or 0% (M = 7) (*p* < 0.000001; f2 = 0.75), and the genotype rankings for 20% ethanol were XYM (M = 20) ≈ XXM (M = 19) > XYF (M = 14) > XXF (M = 11) (*p*(full) = 0.32, f2(full) = 0.01, *p*(3-way only) < 0.000001, f2(3-way only) = 1.71) (simple effects: XYF > XXF, XXM > XXF, XYM > XYF; all *p* < 0.05).

Lastly, the pattern of the ethanol concentration effect on maximum within-bout rate (g liquid/kg BW/h/bout) (Figure 1H) was similar to that of the mean bout rate, as within-bout maxima decreased as ethanol concentration decreased (20% M = 694, 10% M = 638, 0% M = 503) (*p* < 0.000001; f2 = 0.38), while those for the effect of genotype were slightly different. Although XYM again showed a clear dose-response effect (20% > 10%, 20% > 0%; both *p* < 0.05) and XXF again showed greater maxima for 10% than either 20% or 0% (10% > 0%, 20% > 0%; both *p* < 0.05), XXM also showed a clear dose-response effect (20% > 10%, 20% > 0%, 10% > 0%; all *p* < 0.05) and for 20% ethanol, maxima were greater for XYM than XYF (*p* = 0.02).

### 3.2. Within-Session Patterns of Ethanol Consumption

**Figure 2:**
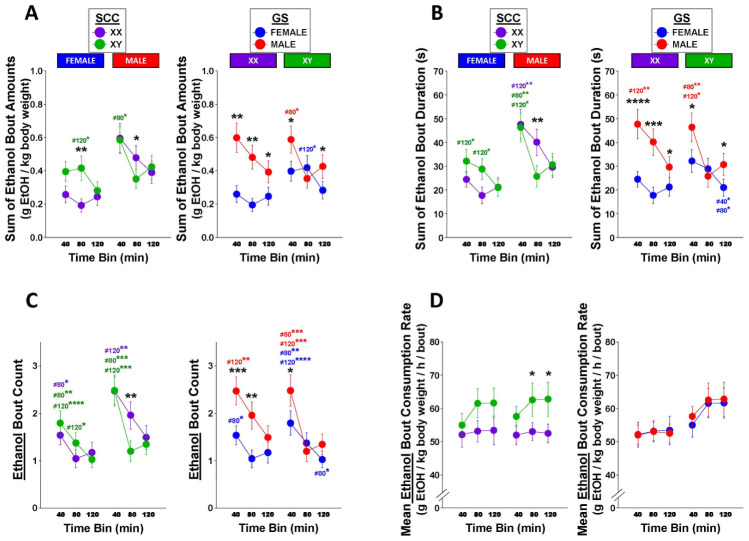
Mean per-session ethanol bout parameters as a function of (within-session) time. (**A**–**D**): Figures plot mean values (error bars represent ±SEM) of time-binned bout parameters for ethanol consumption (20% + 10% ethanol solutions) that are grouped by GS (FEMALE [i.e., with ovaries] or MALE [i.e., with testes]) and split by SCC: XX or XY) (left side of each letter) or vice versa (right side of each letter) so as to better visualize simple effects of GS and SCC within levels of the 2nd factor. These per-bin parameters include the Sum of Ethanol Bout Amounts ((**A**), g/kg), Sum of Ethanol Bout Durations ((**B**), s), Count of Ethanol Bouts (**C**), and Mean Ethanol Bout Consumption Rate ((**D**), g/kg/h; within-bout rates). Sidak-adjusted significance values for simple effects of SCC and GS: * *p* < 0.05, ** *p* < 0.01, *** *p* < 0.001, **** *p* < 0.0001. Sidak-adjusted significance values for simple effects of time bin within genotype use the same asterisk coding for *p*-values, but are color coded to indicate genotype group and numerically indicate the session from which that session differs (e.g., ≠80* in purple represents a significant difference from the 80 min time bin in XX mice).

Across the 2 h session, changes in consumption varied greatly by genotype, with XYF mice consuming more ethanol than XXF mice for only the 1st 80 min, XXM mice consuming more ethanol than XXF mice for the entire session—but to a greater degree earlier than later—and XYM mice consuming more ethanol than XYF mice only at the beginning and end of sessions (Figure 2A–C). GLMM analyses confirmed these within-session patterns across time bins for bout amount (*p* = 0.013, f2 = 0.06), bout duration (*p* = 0.02, f2 = 0.03), and bout counts (*p* = 0.018, f2 = 0.04).

The mean within-bout ethanol consumption rates (g/kg/bin/bout) of XX mice remained similar across the three 40 min time bins (M = 53), while consumption rates of XY mice increased over the last 80 min (M = 62) compared to the 1st 40 min (M = 56), regardless of GS (Figure 2D). Although the SCC*time bin interaction did not reach statistical significance (*p* = 0.21, f2 = 0.02), the SCC*GS*time bin interaction did reach significance in the three-way only model (*p* = 0.02, f2 = 0.18).

At the group level, all genotypes preferred (% ratio of sums of bout amounts) the ethanol solutions (20% + 10%) to water (0%) (M = 73%); with similar ethanol preferences of 20% alone over 0% (M = 65%) and 20% over 10% (M = 61%). The overall ethanol preference (20% + 10% vs. 0%) was higher in males (M = 76%) than females (M = 71%) (*p* = 0.03, f2 = 0.04), regardless of SCC. Similarly, preference for 20% over 0% was greater in males (M = 70%) than females (M = 60%) (*p* = 0.01, f2 = 0.05), and preference for 20% over 10% was greater in males (M = 65%) than females (M = 57%) (*p* = 0.03, f2 = 0.04). However, GS and SCC interacted on preference for 20% over 0% (*p* = 0.045, f2 = 0.07) as XXM (M = 73%) showed higher preference than XXF (M = 57%) (*p* < 0.01), but no other simple effects were confirmed (all *p* > 0.16). Similarly, GS and SCC interacted on preference for 20% over 10% (*p* = 0.03, f2 = 0.04) such that preference was higher in XXM (M = 67%) than XXF (M = 53%) (*p* = 0.001), but there were no other simple effects (all *p* > 0.11).

### 3.3. BECs vs. Ethanol Consumption

**Figure 3:**
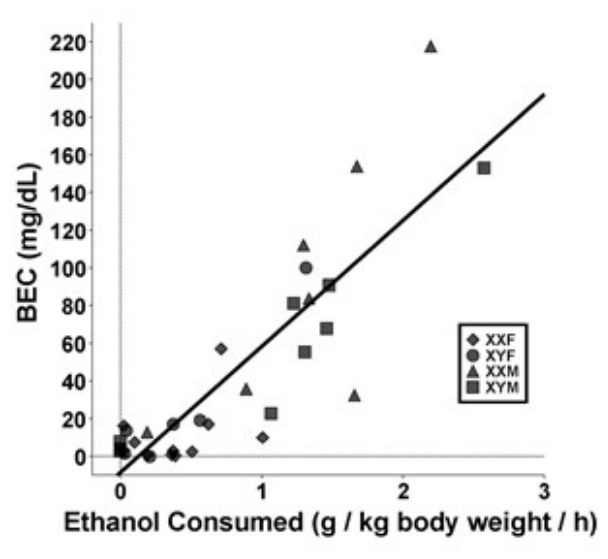
BECs as a function of ethanol consumed. Blood samples were collected within 5 min of the completion of the last session; and BECs (mg/dL) are plotted against ethanol consumption (g/kg BW/h). No statistically significant main effect of either GS or SCC, nor of their interaction, was found for BECs (ANCOVA, all *p* > 0.13), but there was a main effect of EtOH (*p* < 0.000001) (Spearman’s rho for BEC vs. EtOH: rs(33) = 0.79). The equation for the line is Y(BEC) = −8.4 + 67 × X(EtOH). Group n values for BECs: XXF = 11, XXM = 7, XYF = 6, XYM = 9.

Post-session BECs (in mg/dL: M = 42, MAX = 217, MIN = 0) were positively correlated to within-session ethanol consumption (sum of ethanol bout amounts: g/kg/h) (rs(33) = 0.79, *p* < 0.000001) (Figure 3). ANCOVA confirmed the effect of ethanol consumption on BEC (*p* < 0.000001, f2 = 2.21), but no effects of SCC or GS (all *p* > 0.13).

## 4. Discussion

### 4.1. SCC and GS Interact on Ethanol Binge Drinking in Adolescent Mice

In contrast to many ethanol-drinking studies in rodents—particularly DID experiments in wild-type mice—in which females (XX with ovaries) typically consume more ethanol than males (XY with testes), our results obtained using a modified three-bottle DID procedure in adolescent FCG mice reveal an interaction between SCC and GS. Specifically, gonadal males consumed more ethanol than gonadal females, with this difference being greater in XX than in XY mice, and XY mice consumed more ethanol than XX mice only among gonadal females. Notably, consumption was the lowest in XXF mice. These results are consistent with sex differences in ethanol consumption in humans, as historically males typically drink more than females across age (in years) groups (adults (35–45) [96], (18–97) [97], young adults (21–30) [8], (18–24) [21], adolescents (13–17) [3], (14–19) [7], (13–21) [2], and even children (≤12) [98]), a difference potentially increasing with developmental age [5,34,99], but decreasing across generations [5,35,36,37].

Our findings of GS (and SCC) differences in ethanol consumption could be due to one or more procedural differences between our three-bottle procedure and studies using one-bottle and/or two-bottle procedures. These may include the obvious difference—the number of bottles/ethanol concentrations available—and/or the use of adolescent mice (28–31 days old) rather than the more common use of adult mice (>60 days old), the particular characteristics of FCG mice, and the specifics of daily testing (any of the routine procedures involved in daily testing that vary across laboratories).

As in the original Rhodes et al. DID procedure [64], many researchers reporting on sex effects on ethanol drinking provide access to only one bottle during a DID session—a bottle of ethanol that replaces the water bottle [64,70,73,74,76,77,78,79,82,83,84]. This one-bottle procedure is preferred in studies for which achieving high BECs is more important than determining ethanol preferences by way of a ≥two-bottle procedure [72,80,81,100]. Rhodes et al. found higher BECs, but not necessarily higher ethanol intakes, for the one-bottle drinking procedure compared to the 2-bottle procedure in eight inbred mouse strains (including C57BL/6J) [67].

When sex differences have been reported in studies using one-bottle and two-bottle DID procedures, it has most commonly been found that females drank more ethanol than males, while differences in preference and/or achieved BECs were less consistent. As ethanol consumption is normalized to body weight, and females weigh less than males, this pattern may indicate that these sex differences in consumption are influenced, in part, by sex differences in body weight. Our results also indicate a modest contribution of body weight, as effect sizes for the GS*SCC interaction on ethanol consumption amounts (20% + 10% or 20% alone; normalized to body weight) were larger than those for durations (not normalized to body weight). However, not only are GS differences in body weight smaller in adolescence than adulthood, but for the FCG mouse in particular, XX mice weigh more than XY by PD45 [101,102], a difference that emerges by postnatal day 25 [103]. Our results have confirmed this body weight pattern in adolescent FCG mice (postnatal days 28–31). Thus, although the SCC effect on ethanol consumption amounts in gonadal females (XY > XX) is in the same direction as that of the SCC effect on body weight (smaller body weight denominator in XY than XX), this neither explains the GS*SCC interactions with ethanol consumption amounts (males > females; larger effect size in XX than XY mice), nor the similar (though smaller) GS*SCC interactions with durations. Thus, as the GS and SCC differences in ethanol consumption reported herein cannot be fully explained by differences in body weight, they more likely reflect group differences in ethanol pharmacokinetics and/or pharmacodynamics.

Interestingly, a 2015 report by Barkley-Levenson and Crabbe, using a line of adult mice selected for high-DID BECs (HDID, derived from HS/Npt), compared one-bottle to two-bottle DID procedures and did not detect a sex difference in total ethanol consumption using the one-bottle procedure (but, there were sex differences in bout number—male > female—and bout duration—female > male; indicating different patterns of consumption), but did detect a sex difference using the two-bottle procedure; the latter was similar to that which we report (males > females) [68]. Additionally, although the study of ethanol drinking in FCG mice by Sneddon et al. used 24 h sessions and 5-day testing blocks of escalating ethanol concentration, the effects on ethanol consumption and preference of both GS (females > males) and SCC (XX > XY) were concentration dependent [20].

Lastly, studies that used scheduled high alcohol consumption procedures [104] in C57BL/6J mice reported greater ethanol intake in females compared to males in both adolescence and adulthood when either 2 h or 24 h sessions and ethanol solutions from 5% to 20% were used [105], but greater ethanol intake was observed in adolescent, but not adult, males compared to females on a version using intermittent 30 min sessions and a 5% ethanol solution [106]. Thus, changes to ethanol concentrations, session scheduling/duration, age of mice and other environmental variables can potentially reverse the anticipated direction of sex differences in ethanol consumption. Considering the importance of resolving challenges to the construct and external validity of preclinical models of voluntary binge-like ethanol consumption, like DID (e.g., by determining what factors increase face validity with the ethanol consumption patterns in humans), further studies on the relationship between the aforementioned procedural variations and GS/SCC differences are vital.

However, regardless of the factors that moderate and/or mediate sex differences in models of binge drinking, our microstructural analyses of consummatory behavior further strengthen the construct validity of our procedure. Specifically, most of the ethanol consumption occurred early in the session (i.e., there was “front loading”), was completed in a single, or very few, bouts (bout maxima were only slightly smaller than bout sums), and for the 20% ethanol solution, consumption rate within a bout was ~80 g EtOH/kg/h. Moreover, this front-loaded within-session pattern of ethanol consumption was sufficient to maintain intoxicating BEC levels at the end of a final 2 h session in approximately ¼ of the subjects.

### 4.2. Possible Mechanism of SCC*GS Interaction

General mechanisms by which GS and SCC could have interacted on ethanol consumption in our study are an effect of SCC on pre-adult levels of gonadal hormones and/or responsivity to those hormones (e.g., SCC effects on estrogen and/or androgen receptor expression) or (2) the effects of gonadal hormones being dependent upon SCC (e.g., sex chromosomal differences in available response element binding sequences for the nuclear receptors for estrogens and androgens). However, in the FCG mouse, effects of SCC on gonadal hormone levels within the GS have not been observed in adulthood [85] or in periadolescence [103]. Thus, although prenatal/perinatal studies have yet to be reported, it seems unlikely that the observed interaction was due to SCC effects on hormone levels in early life. Still, there may be other differences in the maturation of sex steroid signaling (e.g., differences in steroid receptor density or signaling) that have yet to be studied or documented.

Other factors may contribute to the SCC by GS interaction reported here. For example, if the organizational or activational effects of a particular sex steroid influence a measured phenotype in the direction opposite to that of those elicited by the sex chromosome karotype typically associated with it, an interaction like that observed here could result. In one example, Y chromosome dosage has been reported to increase anxiety-like behavior, while testosterone was observed to decrease anxiety-like behaviors in FCG mice [107]; this could produce GS effects that depend on SCC. Future studies of ethanol drinking that use gonadectomized FCG mice, with and without replacement of sex steroids, could address this question more directly.

### 4.3. Focus on Genetic Sex-Biasing Factors

Ethanol-drinking phenotypes in mice are substantially affected by heritable factors [67,68,69,72,82,83,108,109,110,111,112]. Some of these genetic contributors could implicate genes expressed from the X and/or Y chromosome and/or interact with X- or Y-linked transcripts to affect outcomes.

*KDM6A* (X-linked Lysine (K)-specific demethylase 6A or UTX) is an H3K27me2/3 demethylase that removes repressive methyl marks on histones to increase expression of genes [113,114]. It escapes X-inactivation in mice and humans and thus is expressed at higher levels in XX relative to XY cells of many tissues, including brain [115,116,117,118]. Accordingly, it is a candidate gene for causing sex differences in disease phenotypes that are sensitive to the number of X chromosomes [119,120,121].

The potential neurobiological functions of other X- or Y-linked genes that could be relevant here remain underexplored. One exception is *Sry*, a Y-linked gene, that is known to be expressed in dopaminergic neurons and to regulate their function [122]. Given the contribution of dopamine to ethanol reinforcement and consumption [123], this gene could also be of interest to SCC effects on drinking.

### 4.4. Limitations

The current study had several important limitations. First, consistent with the general practice in our vivarium facility at the time, mice were fed a diet that contained soy-derived phytoestrogens, which could affect the impact of sex-biasing factors on behavior. Second, mice from this line of FCG mice are now recognized to carry an X->Y genetic translocation that results in nine genes showing atypical patterns of gene expression (XY > XX), complicating the interpretation of SCC effects [124].

## 5. Conclusions

These studies reveal that, in intact FCG mice, both SCC and GS interact to influence ethanol drinking behaviors; these findings add to the literature by documenting the multiple biological factors that likely contribute to observed sex differences in alcohol drinking in adolescent humans. Results from such lines of investigation may provide valuable insights into the interdependency of genetic and gonadal hormone contributions to generating the sex differences observed in adolescent binge drinking, as well as in AUD more generally.

## Data Availability

The datasets generated and analyzed in this study are available from the corresponding author upon reasonable request, due to the fact that they represent a part of a larger ongoing study that addresses distinct hypotheses.

## Abbreviations

The following abbreviations are used in this manuscript:

AICs	Akaike Information Criterion
AUD	Alcohol use disorder
ANOVA	Analysis of variance
BECs	Blood ethanol concentrations
BW	Body weight
DID	Drinking in the dark
FCG	Four core genotypes
GLMM	Generalized linear mixed model
GS	Gonadal sex
SCC	Sex chromosome complement
